# Menin Inhibitors: New Targeted Therapies for Specific Genetic Subtypes of Difficult-to-Treat Acute Leukemias

**DOI:** 10.3390/cancers17010142

**Published:** 2025-01-04

**Authors:** Pasquale Niscola, Valentina Gianfelici, Marco Giovannini, Daniela Piccioni, Carla Mazzone, Paolo de Fabritiis

**Affiliations:** Hematology Unit, S. Eugenio Hospital (ASL Roma 2), 00122 Rome, Italy; valentina.gianfelici@aslroma2.it (V.G.); marco.giovannini@aslroma2.it (M.G.); daniela.piccioni@aslroma2.it (D.P.); carla.mazzone@aslroma2.it (C.M.); paolo.de.fabritiis@uniroma2.it (P.d.F.)

**Keywords:** menin, menin inhibitors, acute myeloid leukemia, genomic mutations, KMT2A, NPM1, HOX, revumenib, ziftomenib, targeted therapy

## Abstract

Menin (MEN1) is a well-recognized powerful tumor promoter in acute leukemias (AL) with KMT2A rearrangements (KMT2Ar, also known as MLL) and mutant nucleophosmin 1 (NPM1m) acute myeloid leukemia (AML). MEN1 is essential for sustaining leukemic transformation due to its interaction with wild-type KMT2A and KMT2A fusion proteins, leading to the dysregulation of KMT2A target genes. MEN1 inhibitors (MIs), such as revumenib, ziftomenib, and other active small molecules, represent a promising new class of therapies currently under clinical development. By disrupting the MEN1-KMT2Ar complex, a group of proteins involved in chromatin remodeling, MIs induce apoptosis and differentiation AL expressing KMT2Ar or NPM1m AML. Phase I and II clinical trials have evaluated MIs as standalone treatments and combined them with other synergistic drugs, yielding promising results. These trials have demonstrated notable response rates with manageable toxicities. Among MIs, ziftomenib received orphan drug and breakthrough therapy designations from the European Medicines Agency in January 2024 and the Food and Drug Administration (FDA) in April 2024, respectively, for treating R/R patients with NPM1m AML. Additionally, in November 2024, the FDA approved revumenib for treating R/R patients with KMT2Ar-AL. This review focuses on the pathophysiology of MI-sensitive AL, primarily AML. It illustrates data from clinical trials and discusses the emergence of resistance mechanisms. In addition, we outline future directions for the use of MIs and emphasize the need for further research to fully realize the potential of these novel compounds, especially in the context of specific genetic subtypes of challenging AL.

## Simple Summary

KTMA2 gene rearrangements (KMT2Ar) frequently occur in acute leukemias (ALs) and portray an aggressive clinical phenotype, a high relapse risk, and a dismal prognosis. In these subsets of AL, the binding of the protein Menin (MEN1) to KMT2A fusion proteins drives the activation of a transcriptional pathway, altering the transcription of various genomic entities, particularly HOX/MEIS1 genes, which are crucial in the development of the leukemic phenotype. In this regard, KMT2Ar is prevalent in acute myeloid leukemia (AML), acute lymphoblastic leukemia, and some rarer AL subtypes. In addition, nucleophosmin-1 (NPM1m) mutations induce a gene expression profile similar to other KMT2Ar ALs, specifically in NPM1m AML. Therefore, inhibiting the interaction between MEN1 and KMT2Ar and NPM1m proteins induces apoptosis and promotes the differentiation of leukemic cells. In this regard, some targeted agents, such as MEN1 inhibitors, have been developed and are in an advanced phase of clinical investigation with encouraging clinical results. They have recently received their first formal recognition from the U.S. and European drug regulatory agencies.

## 1. Introduction

Menin (MEN1) is a well-recognized powerful tumor promoter in acute leukemias (ALs) with lysine methyltransferase 2A (KMT2A, previously known as MLL: mixed-lineage leukemia) rearrangements (KMT2Ar) and mutant nucleophosmin 1 (NPM1m) [1,2,3]. KMT2Ar ALs can present as myeloid, lymphoid, or mixed-lineage clinical phenotypes, whereas NPM1m characterizes a distinct subset of acute myeloid leukemia (AML). MEN1 is essential for sustaining leukemic transformation due to its interaction with wild-type KMT2A-related fusion proteins, leading to the dysregulation of KMT2A target genes [1]. KMT2Ar frequently occurs in ALs with homeobox (HOX) and myeloid ecotropic virus insertion site 1 (MEIS1) gene overexpression [4,5,6,7,8]. The latter genomic abnormalities are crucial in developing the leukemic process [8,9,10,11,12,13,14,15], activating abnormal transcriptional pathways and leading to synthesizing fusion peptides that bind MEN1 protein and promoting the development of the leukemic process. AL subtypes with KMT2Ar portray a poor prognosis, higher rates of relapse, and resistance to chemotherapy [1,2,3,4,15,16,17]. KMT2Ar can appear in acute lymphoblastic leukemia (ALL) [18,19,20] as well as in some rare but difficult-to-treat ALs, such as those harboring upstream binding transcription factor (UBTF) tandem duplications [21,22,23], Nucleoporin 98 (NUP98) rearrangements (NUP98r) [24,25,26,27,28] and mixed phenotypes (MPAL) [29]. However, this genomic abnormality primarily occurs in AML (5–10% of adults) [1,2,3,4]. NPM1m [9,10,11,12,13,14,15,16] defines a distinct subtype of AML with a gene expression profile similar to other KMT2Ar AL [30] and occurs in one-third of newly diagnosed (ND) AMLs in adult patients [12]. In general, AMLs are typically diagnosed at a median age of 70 years and account for 80% of all ALs [31,32]. AML is a myeloid cancer mainly due to epigenetic changes [5,6,7,8,9,10,11,12,13,14,15,16,17,18,19,20] that can impair the ability of clonal hematopoietic cells to self-renew or differentiate [1,2,33,34], resulting in the uncontrolled proliferation of leukemic cells. Indeed, in committed hematopoietic progenitors, aberrant histone modifications significantly derepress self-renewal genes, such as HOXA/B and MEIS1 [1,5]. This mechanism is crucial in developing ALs, particularly with KMT2Ar and NPM1m [5,20], and recent findings are relevant for treating AML. Indeed, advancements in understanding its pathology have led to significant changes in treatment approaches over the past 20 years [31,32,33,34]. The most notable developments have been regarding evaluating the patient’s overall fitness, which has replaced age as the main factor in treatment decision-making [31]. Moreover, the evolution of AML classifications has shifted their frameworks from morphology-based to mutation-based systems, as seen in updates from the fifth risk classification from the World Health Organization (WHO-5) [35], the International Consensus Classification (ICC) [36], and the European Leukemia Net (ELN) [37], which have paralleled advances in treatment options over the last few years. Indeed, insights into the molecular pathobiology of AML have helped identify therapeutic targets and develop novel specifically tailored treatments toward multiple subsets of AML [31,32,33,34,35,36], including several mutation-specific tailored therapies, such as inhibitors of Fms-like tyrosine kinase 3 (FLT3) [38] and isocitrate dehydrogenase (IDH) [39,40]; glasdegib [41], an inhibitor of the hedgehog signaling pathway; and B cell leukemia/lymphoma 2 (BCL-2) inhibitors, such as venetoclax [42,43]. In particular, the therapeutic combination of venetoclax with hypomethylating agents, particularly azacytidine, is now a first-line treatment and the new standard of care in older patients with AML [31]. The therapeutic application of these novel-tailored compounds has significantly improved the outcome of AML in affected individuals unfit for intensive chemotherapy (ICT) and allogeneic hematopoietic stem cell transplant (HSCT), such as older patients [33]. Despite these advancements, some forms of AML and other subtypes of AL are complex to treat [31,33,44,45,46,47,48,49,50]. Additionally, the clinical management of refractory/relapsed patients (R/R) remains a challenging concern [48,49,50]. Therefore, new antileukemic agents are in clinical trials or have recently gained approval, emphasizing the value of targeted therapies based on specific mutations [31,51,52,53]. With this regard, MEN1 inhibitors (MIs) are targeted agents currently in clinical development [1,34,51,52,53,54,55,56,57]. These small active compounds prevent the binding of the MEN1 protein to the KMT2Ar complex. This action inhibits the leukemic pathway, leading to apoptosis and the differentiation of immature leukemic cells [1,2,3,34]. This review discusses the biological properties of MIs and summarizes the current clinical developments regarding their use in treating NPM1m AML and KMT2Ar-specific genetic subtypes of challenging ALs [1,3,6,17]. Additionally, it highlights the potential future applications of these novel and specifically tailored therapeutic tools.

## 2. The Inhibition of MEN1 and the Pathophysiology of MI-Sensitive Acute Leukemias

**Figure 1:**
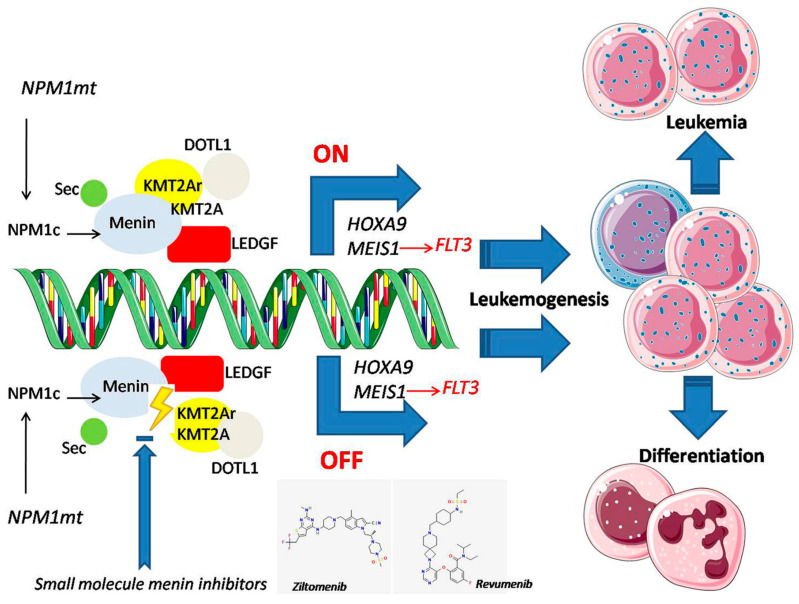
The critical role of MEN1 in regulating gene expression. MEN1 interacts with various transcription factors and chromatin regulators, particularly by binding to KMT2A. This binding site is conserved across all KMT2A fusion proteins and is an essential cofactor for interactions with HOX gene promoters. KMT2Ar leukemias are characterized by the abnormal overexpression of HOX genes and their cofactor, MEIS1. In contrast, NPM1m is primarily located in the cytoplasm and exhibits a gene expression profile that resembles that of KMT2Ar leukemias, featuring the upregulation of HOX genes. This results in a block of hematopoietic differentiation and contributes to leukemic transformation. Revumenib and ziftomenib are MEN1 inhibitors disrupting the chromatin complex between MEN1 and KMT2A. By inhibiting this interaction, these inhibitors target the abnormal transcriptional program linked to leukemogenesis and induce apoptosis without adversely affecting normal hematopoiesis [34]. Legend: KMT2A: Lysine Methyltransferase 2A; NPM1: Nucleophosmin 1; AML: Acute Myeloid Leukemia; HOX: Homeobox Gene Family; MEIS1: Meis Homeobox 1; SEC: Super Elongation Complex; DOT1L: DOT1-Like Histone Lysine Methyltransferase; LEDGF: Lens Epithelium-Derived Growth Factor (Taken and adapted with author’s permission from reference [34]).

MEN1, a nuclear scaffold protein, is a key player in various biological processes, such as hematopoiesis and myeloid proliferation. MEN1 functions as a transcriptional co-regulator; it interacts with transcription factors but does not bind directly to DNA. Notably, its interactions with the KMT2Ar protein are essential for the leukemic transcriptional program in ALs, as these interactions lead to the upregulation of HOX/MEIS1 genes [13,30,31,32,33,34,35,36] (Figure 1) [34]. Mutations in MEN1 result in a loss of MEN1 function, contributing to cell proliferation and tumorigenesis. The KMT2A proto-oncogene is an essential epigenetic regulator in hematopoiesis [1]. KMT2A’s biological activity consists of binding to more than 80 fusion partners, most of which are transcriptional cofactor proteins. In KMT2Ar AL, these fusion proteins bind MEN1, interacting with chromatin-associated protein complexes [20]. KMT2A is a crucial promoter in enhancing the oncogenic properties of the HOXA/B, MEIS1, and FLT3 genes, whose expression regulates transcription through epigenetic mechanisms, promoting the proliferation and differentiation of hematopoietic cells. Notably, HOX A/B and MEIS1 expression levels are high in immature stem cell populations but decline during the maturation and differentiation of hematopoietic cells. Consequently, the interaction between KMT2Ar fusion proteins and MEN1 is crucial for developing KMT2Ar AL. Once MEN1 binds, KMT2A fusion proteins relocate to the nucleus, triggering the abnormal transcription of the HOXA gene and other critical genes, such as MEIS1, for the pathogenesis of KMT2Ar Als [1,13,33,34,35]. In contrast, when MEN1 binding is lost, the oncogenic properties of KMT2A fusion proteins are abolished in KMT2Ar AL [28,29,30,31,32,33,54,55,56,57,58,59]. However, recent findings by Florian Perner et al. show that KMT2Ar cells remain dependent on KMT2A fusion proteins, even after losing MEN1 dependency [60]. Point mutations in the MEN1 gene during drug treatment can lead to resistance against MEN1 inhibitors, significantly contributing to treatment failure. The authors investigate the phenomenon of acquired, non-genetic resistance to MEN1 inhibitors (MIs) and identify an epigenetic switch that may potentially reprogram resistant cells back into a sensitive state. Their findings suggest that non-genetic adaptation processes could play a role in the treatment evasion of leukemic cells. MEN1 connects transcription factors and epigenetic effectors, contributing to its function as an oncogenic tumor promoter in KMT2Ar AL, NPM1m AML, and other rare AL subtypes associated with poor outcomes [60]. Among MI-sensitive Als, NPM1m AML represents a distinct disease entity in 2022 WHO-5 [35] and ICC/ELN classifications [36,37]. NPM1 is a nuclear chaperone protein that shuttles between the cytoplasm and the nucleus, performing various biological functions under normal circumstances and regulating cell growth and DNA repair. The most common NPM1 gene abnormalities occur through insertions in exon 12, specifically A and B, leading these mutations to the stable delocalization of the protein into the cytoplasm. This delocalization is a crucial step in the leukemic transcriptional program, which depends on MEN1 and MEIS1. Mutations in the NPM1 gene are commonly found in AML, occurring in nearly one-third of ND cases. Patients with NPM1m may experience variable clinical courses influenced by other cytogenetic and molecular abnormalities. Mutations in NPM1, always heterozygous, are a critical factor in leukemia. These mutations result from four-base pair insertions, which cause a frameshift mutation at the C-terminus. This leads to the loss of tryptophan and the formation of a new nuclear export signal (NES). As a result, there is a shift towards nuclear export and the cytoplasmic localization of the NPM1m protein. The interaction with exportin 1 (XPO1) facilitates this transfer out of the nucleus [9,10,11,12,13,14,15]. XPO1, previously known as Crm1p, is a nuclear transporter acting as a direct carrier and mediating the export of NES proteins into the cytoplasm. NPM1m is associated with a high HOX gene expression and its cofactors MEIS1 and PBX3, and it is a key factor in the increased self-renewal of leukemic clones. Almost all mutations in NPM1 are confined to exon 12; however, in rare cases (less than 1%), they can also involve exon 6, exon 9, and exon 11. The detection of NPM1m can be performed using molecular tests, such as next-generation sequencing and immunohistochemistry [12,15]. The aberrant cytoplasmic dislocation of NPM1m can be considered the critical initial step in leukemogenesis, resulting in significant cellular derangements, including uncontrolled centrosome duplication, the inhibition of tumor suppressor genes, enhanced proteolytic activities of caspases 6 and 8, defective DNA repair mechanisms, and the activation of the MYC oncogene [9,10,11,12,13,14,15]. Animal studies indicate that this event by itself may not always lead to full-blown disease, implying that a second mutation may be necessary for the progression to overt leukemia [12]. At diagnosis, patients with NPM1m AML typically present with a high percentage of blasts, elevated white blood cell and platelet counts, and increased extramedullary involvement. This variant is often associated with a normal karyotype. A minority of patients may exhibit additional minor secondary chromosomal abnormalities, most commonly being +8, +4, deletions on 9q, and +21. About 15% of patients may have an abnormal karyotype, but these abnormalities do not have prognostic implications for overall survival (OS) [12]. NPM1m is restricted to myeloid cells and is not found in B and T cells in peripheral blood or bone marrow (BM). While NPM1m has been noted in chronic myelomonocytic leukemia, these cases typically progress to AML shortly after diagnosis. Unlike mutations associated with clonal hematopoiesis (DNMT3A, TET2, and SF3B1), NPM1m is the ’first hit’ of the leukemic process. Although the exact mechanisms by which NPM1m contributes to AML still need to be fully understood, in NPM1m AML, the wild-type MEN1-KMT2A complex directly interacts with highly expressed levels of HOXA and MEIS1, paralleling the genetic profile observed in KMT2Ar AL [8,12,34]. However, the emergence of novel agents targeting XPO1 and HOX, which influence the intranuclear relocation of mutated NPM1, offers new hope in treating this AML subtype [9,10,11,12,13,14,15,51,57,61,62,63]. However, MEN1 may also have leukemogenic roles independent of its interactions with KMT2Ar [21,22,23,24]. Indeed, MEN1 is involved in the Wnt/β-catenin signaling pathway and regulates chromatin remodeling [64]. It works alongside histone methyltransferases, like KMT2A, which are critical in developing AL. Indeed, MEN1 can suppress tumorigenesis by inhibiting Wnt/β-catenin signaling, a pathway involving a family of proteins crucial for embryonic development and maintaining adult tissue homeostasis. In vitro studies have shown that the absence of MEN1 results in an increased nuclear accumulation of β-catenin, which subsequently enhances cell proliferation. The Wnt pathway is a crucial cell proliferation, survival, differentiation, and migration regulator. It can be activated when extracellular Wnt ligands, such as R-spondin, syndecan-1, Norrin, and ADNP, bind to membrane receptors. In contrast, other molecules can exert inhibitory effects on the Wnt pathway [64]. Once activated, the Wnt pathway stabilizes β-catenin, allowing it to translocate to the nucleus, promoting gene expression in AL development. MEN1 directly interacts with β-catenin, promoting its export from the nucleus through the OXP1/CRM1 pathway. This pathway is responsible for regulating the nucleocytoplasmic transport of various cargo proteins, including MEN1 itself. As a result, MEN1 inhibits Wnt/β-catenin signaling. This suggests that the pharmacological inhibition of this pathway may be a promising therapeutic approach [1,34,64]. Indeed, inhibiting XPO1 can cause MEN1 to accumulate in the nucleus. Selective inhibitors of nuclear export (SINE) covalently bind to cysteine 528 in the binding pocket of XPO1, disrupting nuclear export and accumulating MEN1 in the nucleus. In addition to interacting with signaling pathway modulators like Wnt/β-catenin, MEN1 may contribute to leukemogenesis in various ways independent of KMT2A rearrangements [1]. MEN1 is associated with chromatin and the nuclear matrix, where it directly binds to double-stranded DNA (dsDNA) and regulates gene expression. Therefore, its localization within the nucleus is essential for gene transcription regulation [65]. MEN1’s key interaction partners include transcription regulators, epigenetic modifiers, chromatin regulators, and DNA damage response proteins [1,2,3,55,56,57]. Additionally, MEN1 can interact with various target genes involved in leukemogenesis different from KMT2A, also regulating pro-leukemogenic genes HOXA9 and MEIS1, such as RUNX1. In this regard, MEN1 enhances its transcriptional activity and activates RUNX1 target genes, contributing to leukemic transformation [1,55,57]. Another proto-oncogenic transcription factor, MYC [66], is a partner binding of MEN1, and their interaction is crucial for enhancing the transcription of MYC target gene expression in this context. Furthermore, MEN1 is linked to chromatin and the nuclear matrix, playing a vital role in DNA damage repair by accumulating more in the nuclear matrix [1,55,56,57].

## 3. Menin Inhibitors: Investigated Agents and Ongoing Clinical Trials in AML

Targeting MEN1 has emerged as a promising therapeutic strategy for AL, given that MIs may disrupt AL cells’ survival and proliferation signals, ultimately leading to their differentiation and apoptosis. Current research focuses on small molecules inhibiting the MEN1-KMT2Ar interaction [1,2,3,23,24,25,26,27,28,29,30,31,32,33,34,67,68,69,70], such as MIs, which can act alone or in association with other treatments [71,72,73,74,75,76,77,78,79] to enhance effectiveness and counteract resistance mechanisms [80,81,82]. Moreover, to overcome resistance to MIs correlated to MEN1 mutations, a new generation of small-molecule inhibitors, such as JNJ-75276617 (bleximenib) [83,84], the covalent compound BMF-219 (icovamenib) [85,86] (among others) and the more recently synthesized BTC-86 [87] are under active preclinical and clinical evaluation. Among different MIs, two—revumenib [70,88,89,90] and ziftomenib [91,92]—have the most advanced preliminary clinical data. These molecules selectively inhibit the binding of MEN1 to KMT2Ar, resulting particularly effectively in the treatment of KMT2Ar and other AL subtypes where MEN1 is critical and providing significant clinical benefits for R/R patients with AL, for which limited therapy options exist [1,2,3,34,93,94,95,96]. Conversely, their clinical activity is likely lacking or limited in AL subtypes not involving KMT2Ar. Several trials have explored or are currently investigating the safety and efficacy of MIs in KMT2Ar AL or NPM1m AML. Table 1 shows ongoing clinical trials evaluating MIs in AL, while revumenib and ziftomenib agents, as well as other MIs, and their use in clinical combinations to treat distinct subsets of ALs will be addressed explicitly in the following sections.

## 4. Revumenib

The first human trial of revumenib was a phase I/II study called AUGMENT-101 [88]. This multicenter, open-label, dose-escalation trial focused on patients with relapsed or refractory acute leukemia (R/R AL). Initially, the trial observed a lack of clinical activity in patients who did not have either KMT2Ar AL or NPM1m AML. As a result, the protocol was revised to enroll patients with these genetic abnormalities specifically. The study utilized a split-dose escalation design with two arms: one for patients not taking potent CYP3A4 inhibitors and another for those who were. This adjustment was critical due to the significant effects the CYP3A4 pathway has on the metabolism of many drugs, including revumenib. Early pharmacokinetic studies revealed substantial differences in drug metabolism among patients receiving antifungal agents such as posaconazole or voriconazole, known as potent CYP3A4 inhibitors. There were 94 patients, most of whom had R/R AML, with a median age of 37. Participating patients received a median of four prior therapies, and 46% relapsed after allogeneic HSCT. The ORR in the cohort of evaluable patients (57) was 63%, with 23% achieving CR or CR with partial hematologic recovery (CRh). The median CR+CRh duration was 6.4 months. Of the 22 patients who achieved CR or CRh, the median time to reach CR or CRh was 1.9 months, varying from 0.9 to 4.9 months. Among those who achieved CR or CRh, MRD was undetectable in 78%. The median overall survival (OS) and response duration for treated patients were 7 and 9.1 months, consistent with existing preclinical insights into KMT2Ar AL and NPM1m AML pathophysiology. Moreover, among the 83 transfusion-dependent patients, 12 (14%) became transfusion-free. Transcriptional studies using RNA sequencing have revealed that several leukemogenic genes, including HOXA and MEIS1, are downregulated, while genes associated with differentiation are upregulated. Notably, many patients with KMT2Ar who achieved morphological remission after one treatment cycle still showed evidence of KMT2A fusions. In certain instances, multiparameter flow cytometry (MPFC) revealed damaging MRD before cytogenetic normalization, suggesting that this response pattern may indicate a differentiation process in which blast cells gradually mature while retaining cytogenetic abnormalities. As a result, MPFC no longer recognizes these cells as blasts. Of note, these findings highlight the potential for MRD assessment to reshape the future of AL therapy [70,89,90] for patients treated with these novel therapies. The safety profile was predictable, and the side effects were manageable (see above the paragraph on MI safety). Therefore, the impressive clinical results that emerged from a significant evaluation of this targeted therapy led to the FDA approval of revumenib in November 2024 to treat adult and pediatric patients with R/R AL with a KMT2A translocation (www.fda.gov, accessed 20 December 2024).

### 4.1. Ziftomenib

Ziftomenib (KO-539) is an oral medication administered once daily in 28-day cycles. It generates at least two active compounds and shows intense, selective clinical activity as a monotherapy in patients with R/R KMT2Ar ALs and NPM1m AML. This agent induces significant myeloid differentiation effects and exhibits notable antileukemic activity in these contexts. Ziftomenib targets the interaction between MEN1 and the KMT2A protein, acting as a selective inhibitor of the KMT2Ar AL of NPM1m AML. By inhibiting this interaction, ziftomenib disrupts the oncogenic processes that contribute to the progression of AL, serving as an ‘epigenetic’ treatment that modifies gene behavior without altering the DNA sequence. In the KOMET-001 trial, adults with R/R AML participated in a dose-escalation, dose-validation, and expansion (phase 1b) study [91,92]. During phase 1a, patients with all molecular subtypes received ziftomenib orally at doses ranging from 50 to 1000 mg once daily over 28-day cycles. In phase 1b, patients with NPM1m AML or KMT2Ar AL were randomized into two parallel dose cohorts: one received 200 mg while the other received 600 mg of ziftomenib. A total of 83 patients were treated, with a median follow-up time of 22.3 months. In phase 1b, patients who received the 200 mg dose did not achieve any response. However, at the phase 2 dose of 600 mg, 9 out of 36 patients (25%) with KMT2Ar AL or NPM1m AML achieved CR or CRh. Among 20 patients with NPM1m AML treated at this dose, seven (35%) attained CR. Overall, 68 out of 83 patients experienced severe adverse events, including two treatment-related deaths: one due to differentiation syndrome (DS) and the other from cardiac arrest. The careful consideration of patient safety was evident in the decision to stop enrolling patients with KMT2Ar due to the DS rate and severity. Despite these challenges, phase 1b demonstrated promising clinical activity with manageable toxicity in heavily pretreated R/R AML patients, showcasing one of the highest activity levels for any monotherapy in NPM1m patients [12,73,93,94,95]. The phase I/II KOMET-001 [92] trial reported an ORR of 40% and a CR rate of 35% among AML patients with NPM1m. However, outcomes were less favorable for patients with KMT2Ar, revealing an ORR of only 16.7% and a CR rate of 11%. In January 2024, Ziftomenib received orphan drug designation from the European Medicines Agency (EMA). In April 2024, it gained the first breakthrough therapy designation from the Food and Drug Administration (FDA) as a specific therapy for R/R patients with NPM1m AML. These promising clinical results have prompted the initiation of a phase 2 study in patients with NPM1m AML, as well as phase 1 studies evaluating ziftomenib in combination with immune checkpoint therapies and other targeted therapies for NPM1m AML and KMT2Ar ALs. This novel treatment targets a critical pathway involved in AML, providing hope for patients facing this challenging disease. However, additional data are necessary before ziftomenib can be an established treatment option.

### 4.2. Other Menin Inhibitors Under Investigation

Several other investigational MIs, including JNJ-75276617, are currently being evaluated in phase 1 clinical trials [34,54,57,83,84]. Recently, preliminary results from a phase 1 study involving adults with R/R KMT2Ar ALs and NPM1m AML have emerged. The JNJ-75276617 agent, bleximenib, is a potent, orally bioavailable, selective inhibitor of the interaction between MEN1 and KMT2Ar. In cells with KMT2Ar and NPM1m, bleximenib disrupts the association between the MEN1-KMT2A complex and the chromatin at the promoters of target genes. This disruption reduces the expression of several target genes related to MEN1-KMT2A, including MEIS1 and FLT3. Additionally, preclinical studies have shown that bleximenib exhibits significant antiproliferative activity in various AML and ALL cell lines and patient samples with KMT2A or NPM1 alterations when tested in vitro. A phase 1 study evaluating bleximenib included 58 adult patients with R/R ALs (AML:97%; ALL:3% of participants), reporting manageable adverse effects. A notable 63% ORR highlighted the clinical efficacy of this treatment. Notably, at the highest dose tested, the ORR was 50%, with a time to response ranging from 1.0 to 3.3 months [84]. Importantly, JNJ-75276617 has shown significant antiproliferative activity in patients with acute leukemia (AL) who have acquired MEN1 mutations and are resistant to the MEN1-KMT2A inhibitor revumenib. The co-crystal structure of MEN1 complexed with JNJ-75276617 reveals a distinct binding mode different from that of other MEN1-KMT2A inhibitors, including revumenib. Therefore, JNJ-75276617 is currently undergoing clinical investigation for its efficacy in treating R/R ALs that have KMT2Ar or NPM1m as a monotherapy and in combination with therapies specifically targeting AML (Table 1). The initial findings are promising, indicating an acceptable safety profile and antileukemic activity when used as monotherapy, which aligns with results from other MIs [83,84]. Bleximenib is undergoing clinical investigation to treat AL with KMT2A or NPM1 alterations as a standalone therapy for R/R AL and in combination with other targeted therapies [75,76,77,83,84]. Additionally, BMF-219, which is the first and only covalent MI in clinical development, is being evaluated in the COVALENT-101 study. In this study, which included adults with R/R AL who are ineligible for ICT, this agent was tolerable, with no reported dose-limiting toxicities or treatment discontinuation due to related side effects [85,86]. BMF-219 is being tested in adult patients with KMT2Ar AL or NPM1m AML and other hematological malignancies as part of the COVALENT-101 trial (Table 1). A preliminary report included twenty-four AML and two ALL patients, with a median of four prior lines of therapy. Eleven of them had previously undergone allogeneic HSCT. BMF-219 has been well tolerated, with no recorded dose-related toxicities or treatment discontinuations. However, DS was observed in 13% of the cases. Among MIs, DSP-5336 (enzomenib) [54,55,56,57,87] is a small molecule undergoing phase I/II clinical trials, specifically the DSP-5336-101 trial (Table 1). This trial, which enrolls dose-expansion cohorts for adult patients with R/R KMT2Ar ALL or NPM1m AML, is an ongoing journey of discovery. Preliminary results have shown promise. Thirty-five patients with KMT2Ar or NPM1m who had not previously received MIs were treated with active doses of enzomenib. Among the 22 patients with KMT2Ar (20 AML, 2 ALL), the ORR was 59.1%, with a CR or Cri of 22.7%. For the 13 patients with NPM1m AML, the ORR was 53.8%, with a CR or CRi of 23.1%. The median time to CR or CRi was 1.0 months, and importantly, no unmanageable side effects or dose-limiting toxicities were observed. There were no treatment-related discontinuations, treatment-associated QT prolongation, or other cardiac side effects. Notably, DSP-5336 did not cause clinically significant DS [97]. Other MIs have effectively reduced leukemic cell growth and promoted differentiation in preclinical models of AL with both KMT2Ar AL and NPM1m AML. These MIs are undergoing clinical trials, and preliminary findings indicate promising safety profiles, efficacy, and tolerability for these novel compounds [1,54,55,56,57,63].

### 4.3. Menin Inhibitor Combination Therapies and Alternative Targets Within Leukemic Pathways

Many investigator-initiated trials explore combinations involving MIs (as shown in Table 1) to address clinical needs beyond resistance to MEN1. There are resistance mechanisms to venetoclax-based therapies for AML, which is the current standard of care for older patients or those who are unable to receive intensive chemotherapy. Refs. [11,12] revealed the significant activation of a KMT2Ar-like signature [1,17,18,19,20]. Consequently, inhibiting MEN1 may help downregulate HOX and MEIS1, potentially overcoming resistance to venetoclax in AML. These findings open the door to investigating combinations of MIs and venetoclax [1,74,75,76]. One trial, KOMET-007, evaluates the safety, tolerability, and preliminary antileukemic activity of ziftomenib in combination with venetoclax and azacitidine [76], as well as venetoclax with the 7 + 3 regimen, in the management of R/R patients with NPM1m AML or KMT2Ar AL [78]. In this context, ziftomenib combined with venetoclax showed the most promising results as a treatment option for acute leukemia patients with KMT2Ar or NPM1m AML who have not responded to venetoclax-based therapies [76]. An updated report on this study presents very encouraging results. The median age of the 34 participants was 56 years (ranging from 23 to 86), with 50% being female. Among the participants, 41% (14 out of 34) had NPM1m AML, while 59% had KMT2Ar AL. The median follow-up period was 35 weeks for patients receiving 200 mg and 14 weeks for those receiving 400 mg, both within the R/R group with AML harboring NPM1m. The median follow-up for KMT2Ar patients with AL was 15 weeks for both dosage levels. The participating patients in this clinical investigation had received a median of two (1–8) previous therapies. Notably, 32% (11 out of 34) had undergone prior allogeneic HSCT, and 74% (25 out of 34) were naïve to MIs, including 68% (17 out of 25) who had prior exposure to venetoclax. No dose-limiting toxicities (DLTs) or ziftomenib-induced QTc prolongation occurred. On-target DS was observed in four patients (12%) in the R/R group, including one patient with NPM1m AML at the 400 mg dosage and three with AL KMT2Ar. However, all cases of DS were manageable with standard interventions. Among the 24 MI-naïve patients (NPM1m: n = 11; KMT2Ar: n = 13) who underwent at least one response assessment, the ORR for R/R NPM1m AML patients was 100% (five out of five) at 200 mg and 67% (four out of six) at 400 mg. The CR and CRh rates were 80% (four out of five) at 200 mg and 50% (three out of six) at 400 mg. For NPM1m AML patients with prior venetoclax exposure, the ORR was also 100% (three out of three) at 200 mg and 50% (two out of four) at 400 mg. In R/R KMT2Ar patients, the ORR was 43% (three out of seven) at 200 mg and 33% (two out of six) at 400 mg, with CRh rates of 29% (two out of seven) at 200 mg and 17% (one out of six) at 400 mg. For KMT2Ar AML patients with prior venetoclax exposure, the ORR was 40% (two out of five) at 200 mg and 25% (one out of four) at 400 mg. These promising clinical results indicate that the combination of ziftomenib with venetoclax and azacitidine has been well-tolerated at the tested dose levels and continues showing in R/R patients encouraging clinical activity that was remarkable in previously venetoclax-exposed patients [74]. Regarding the therapeutic combination of ziftomenib with ICT, a recent report has updated this investigational experience [76]. The study enrolled adults aged 18 and older with ND NPM1m AL or KMT2Ar AML at high risk. Participating patients were separated into different dose-escalation cohorts according to their genotype. The participating patients had either NPM1m or KMT2Ar AL and received ziftomenib. This medication was administered orally at escalating doses of 200 mg, 400 mg, or 600 mg once daily, in combination with cytarabine and daunorubicin (7 + 3 regimen), starting from the induction phase through to consolidation and subsequent treatments, including the post-transplant phase. The median age of the 35 evaluable patients was 58, ranging from 28 to 75 years. Importantly, there were no reports of drug syndrome (DS), ziftomenib-associated QTc prolongation, or DLTs at the 200 mg or 400 mg dose levels. For patients with NPM1m AML, the composite complete remission (CRc: CR + CRh) was 100% (eight out of eight) at the 200 mg dose and 86% (six out of seven) at the 400 mg dose, with MRD negativity among responders, being 100% (eight out of eight) and 80% (four out of five), respectively. Additionally, patients with KMT2Ar AL achieved a CRc of 90% (nine out of ten) at the 200 mg dose and 63% (five out of eight) at the 400 mg dose; remarkably, the treatment resulted in MRD negativity in 83% and 100% of responders at these respective doses [76]. The SAVE study also examined the efficacy of combining all oral treatments, including revumenib with decitabine/cedazuridine and venetoclax, for patients with R/R AML or MPAL. Preliminary results show an acceptable safety profile and impressively high efficacy, with seven out of eight patients achieving morphological remission and three achieving MRD negativity [79]. There is a rationale for combining FLT3 and MIs due to the downregulation of FLT3 transcription in patients with NPM1m AML who are treated with MIs. Inhibition of the MEN1-KMT2A binding leads to a significant reduction in MEIS1 expression, associated with the downregulation of FLT3. This reduction in FLT3 expression is significant as it indicates the potential efficacy of MEN1 inhibition in AML treatment. Consequently, combining MEN1-KMT2A binding and FLT3 inhibition significantly suppresses STAT5A target genes. The latter is crucial in maintaining AL and is an essential downstream mediator of activating FLT3 mutations [77,98]. Therefore, MIs combined with FLT3 inhibitors can synergistically affect AML models with NPM1m and KMT2Ar. This combination therapy led to a more significant reduction in cell viability than MEN1 or FLT3 inhibitors alone in human AML cell lines with co-mutations in NPM1 and FLT3. Similarly, in mouse xenograft models of KMT2Ar AML with concurrent FLT3 mutations, combining MIs and an FLT3 inhibitor resulted in better survival outcomes than monotherapy with either agent. Thus, combining MEN1 and FLT3 inhibition may improve survival for patients with FLT3-mutated AML with concomitant NPM1m or KMT2Ar. Currently, the field of AML research is actively exploring early-phase combination studies for this patient population. A phase 1 trial of revumenib combined with gilteritinib has recently begun enrolling participants for R/R patients with AML harboring NPM1m or KMT2Ar. The KOMET-008 phase 1 trial included an arm testing the combination of ziftomenib and gilteritinib for FLT3-ITD-mutated R/R AML, showing significant benefits for AML patients with NPM1m or KMT2Ar. Additionally, bleximenib synergizes with gilteritinib in vitro in AML cells that carry KMT2Ar. Moreover, it also shows synergistic interactions with venetoclax and azacitidine in AML cells with KMT2Ar [77,98]. Again, preclinical studies have demonstrated that combining an XPO1 inhibitor with a ziftomenib inhibited KMT2Ar and NPM1m leukemic cells in vitro and in vivo [1,10,12,62,63]. The interaction between NPM1m and XPO1 leads to the abnormal displacement of NPM1m into the cytoplasm. This process results in elevated expression levels of HOX genes, essential for maintaining the leukemic state in NPM1m cells. Therefore, there is a therapeutic rationale for using XPO1 inhibitors. Selinexor was the first XPO1 inhibitor to be clinically tested. However, preliminary results using selinexor as a monotherapy in AML were disappointing [62]. In contrast, the second-generation XPO1 inhibitor, eltanexor [63], has demonstrated the ability to cause an irreversible downregulation of HOX genes, inducing the terminal differentiation of AML cells and prolonging the survival of leukemic mice. Preclinical studies indicated that MIs, such as ziftomenib, combined with selinexor, significantly inhibit the growth of KTA2Ar AML cell lines. In particular, the combination of selinexor and ziftomenib inhibits the MEN1-KMT2A interaction and nuclear export, providing a molecular basis for the synergy observed between the two compounds. Moreover, this treatment association increased apoptosis and reduced MEN1, HOXA9, and MEIS1 protein levels. Therefore, the synergistic effects of the two compounds represent a promising, effective strategy for treating KTA2Ar AML [61]. This preclinical study supports the robust synergy of ziftomenib and XPO1 inhibition beyond *KMT2A*r AL and could be a potential strategy for treating *NPM1m* AML, setting a premise for translational potential [12,63].

## 5. Safety of MIs

Safety assessments revealed that 53% of patients experienced a reversible and manageable prolongation of the QTc interval on electrocardiograms, identified as the only DLT at any grade. Among these patients, 13% had grade 3 or 4 QTc abnormalities. Management strategies were effective in all cases, including electrolyte repletion, withholding revumenib if the QTc interval exceeded 481 msec, and reducing the dosage if the prolongation did not improve within two weeks. It is important to note that all instances of QTc prolongation were reversible, providing a sense of security about the safety of revumenib treatment, and no ventricular arrhythmias occurred. In the AUGMENT-101 phase 1 trial, the most commonly reported adverse events were QT prolongation, which occurred in 56% of patients at any grade. This was followed by nausea in 50% of patients, vomiting in 40%, and febrile neutropenia in 31%. Treatment with revumenib was associated with a low incidence of grade 3 or higher side effects or more severe reactions. Notably, rarely observed asymptomatic prolongations of the QTc interval were identified as the only DLT [88]. Therefore, treatment with revumenib was substantially safe, with a low incidence of severe adverse effects. DS may be a critical drug-induced adverse complication caused by MIs. This syndrome is related to cytokine alterations that occur during hematopoietic differentiation. Symptoms can include fever, joint pain, increased leukocytosis, and, in severe cases, pleural or pericardial effusions, as well as respiratory or renal failure. This syndrome may occur after treatment with IDH inhibitors, arsenic trioxide, or all-trans retinoic acid [1,34,39,99]. DS, which typically developed at a median time of 18 (5–41) days, provides evidence of the successful reversal of the differentiation block caused by KMT2Ar through MEN1 inhibition [88,89,90]. In clinical trials, DS occurred in 16% of patients treated with revumenib [88,89,90] and in 57.5% of patients treated with ziftomenib [76,77,78], with about one-third of cases presenting severe features. In the phase 1 trial with revumenib, all instances of DS resolved after treatment with steroids, and hydroxyurea was added in cases of leukocytosis [76,77,78]. No long-term side effects related to toxicity or the potential development of new tumors in patients who have received MIs have been reported. However, because these new agents have been in use a relatively short time, it is essential to implement the careful long-term monitoring of their effects through routine and thorough patient evaluations.

## 6. Resistance to MIs

As with other targeted therapies, resistance to MIs may develop over time [1,34,60,80,83,87]. The early identification of resistance mechanisms to MIs is crucial to appreciate patient management, allowing for alternative salvage therapies, whenever applicable, and avoiding unnecessary toxicity by offering other options, such as supportive and palliative care [100], in those unsuitable for continuing to receive active and causal measures. Perner et al. conducted a study on BM samples from patients who participated in phase I of the AUGMENT-101 trial [80], specifically focusing on those who initially responded to revumenib but later experienced a relapse. The researchers identified several distinct somatic mutations in the MEN1 gene, which encodes for MEN1 [80]. Notably, these mutations were absent during diagnosis but emerged during revumenib treatment. Over one-third of evaluable patients who received more than two treatment courses showed clonal expansion, a process where a single cell or group of cells with a specific mutation proliferates, leading to a larger population of cells with the same mutation [80]. While not disrupting the interaction between KMT2A and MEN1 or its oncogenic properties, these mutations did affect the cells’ sensitivity to small-molecule inhibitors. Among the evaluable patients who underwent more than two lines of treatment, over one-third exhibited clonal expansion, the proliferation of a single cell or group of cells with a specific mutation, resulting in a larger population of cells containing that mutation [80], and considerable resistance to various MIs. These findings highlighted the specificity of Revumenib’s on-target activity, suggesting the potential for developing additional MIs less affected by selective treatment pressures [1,67]. So, developing second-generation inhibitors that effectively block KTM2A binding while maintaining efficacy against MEN1 mutations is crucial to overcoming the acquired resistance to first-generation MIs. In such a context, applying basic research techniques in biomolecular engineering for the biochemical characterization and synthesis of new proteins, targeting specific biological activities associated with defined MEN1 mutations, can allow for the development of second-generation MIs [67]. In addition, as recently reported, the Matched Molecular Pair Transformation (MMPT) process, which includes a fluorescence polarization assay, is utilized to create second-generation MEN1-KTM2A inhibitors, such as BTC-86 [87]. This compound shows similar binding activity against wild-type MEN1 protein and MEN1 resistance mutations. BTC-86 has proven to be the most effective inhibitor of MEN1-KTM2A binding when considering all MEN1-acquired mutations, especially compared to other clinical molecules. Furthermore, BTC-86 exhibits excellent pharmacokinetic properties. It also presents a lower risk of QTc prolongation and shows improved metabolic stability in humans compared to first-generation MIs [87]. In conclusion, treatment modalities to overcome resistance to MIs represent an emerging clinical need to address future investigation being this a crucial need due to the ever-increasing role that these agents will have in the therapeutic armamentarium for these difficult-to-treat subsets of AL [1,101].

## 7. Conclusions

MIs are an outstanding example of the development of targeted therapies in specific subtypes of biologically complex ALs featuring treatment difficulty and high lethality [17]. Preliminary efficacy and safety data from phase I/II clinical trials of revumenib in patients with R/R KMT2Ar or NPM1m-associated AL are promising. The rapid therapeutic responses observed were particularly encouraging. Notably, among the responders, there was a high rate of MRD negativity, a strong indicator of the treatment’s effectiveness and a robust indication for fit patients to proceed with allogeneic HSCT as a timely treatment consolidation. Moreover, further research into potential strategies to overcome resistance mechanisms, possibly using second-generation MIs or therapeutic combinations including them, will further optimize the clinical use of MIs. Additionally, an ongoing investigation is exploring the applicability of MIs to other AL subtypes overexpressing HOXA/MEIS1. In this regard, developing a clinically validated assay to measure HOXA/MEIS1 expression could help guide treatment decisions and assess responses in the future. Clinical trials involving other MIs in front-line and maintenance therapy, whether as monotherapies or combined with other measures, are ongoing. Although early clinical data are promising, more research is needed to optimize these novel therapies with MIs [34,67], understand their long-term effects, and determine how best to integrate them into existing treatment regimens, including ICT for ND AML and less-intensive approaches for unfit or older patients. In conclusion, MIs represent a significant scientific advancement by effectively inhibiting a protein–protein interaction within an epigenetic chromatin modifier. This marks a critical clinical milestone as the first targeted therapy for challenging KMT2Ar ALs and NPM1m AML [1,3,56,101].

## Figures and Tables

**Table 1:** Ongoing recruiting trials on menin inhibitors in acute leukemias.

Clinical Study	ClinicalTrials.gov Identifier
A Study of DSP-5336 in Relapsed/Refractory AML/ALL with or Without MLL Rearrangement or NPM1 Mutation.	NCT04988555
SNDX-5613 and Gilteritinib for treating Relapsed or Refractory FLT3-Mutated Acute Myeloid Leukemia and Concurrent MLL-Rearrangement or NPM1 Mutation.	NCT06222580
Testing the Addition of an Anti-cancer Drug, SNDX-5613, to the Standard Chemotherapy Treatment (Daunorubicin and Cytarabine) for Newly Diagnosed Patients With Acute Myeloid Leukemia That Has Changes in NPM1 or MLL/KMT2A Gene.	NCT05886049
Revumenib in Combination With 7 + 3 + Midostaurin in AML.	NCT06313437
A Study of Revumenib in Combination with Chemotherapy for Patients Diagnosed With Relapsed or Refractory Leukemia.	NCT05580861
A Phase 1/2 Study of Bleximenib in Participants with Acute Leukemia	NCT04811560
First in Human Study of Ziftomenib in Relapsed or Refractory Acute Myeloid Leukemia	NCT04067336
Ziftomenib in Combination with Chemotherapy for Children with Relapsed/Refractory Acute Leukemia. A Phase I-II Study Investigating the All-Oral Combination of the Menin Inhibitor SNDX-5613 With Decitabine/Cedazuridine (ASTX727) and Venetoclax in Acute Myeloid Leukemia (SAVE) NCT05360160.	NCT06376162
A Phase I Study Investigating the Combination of the Menin Inhibitor Ziftomenib With Venetoclax and Gemtuzumab in Pediatric Patients with Acute Myeloid Leukemia.	NCT06448013
A Study of BN104 in the Treatment of Acute Leukemia.	NCT06052813
Study of BMF-219, a Covalent Menin Inhibitor, in Adult Patients With AML, ALL (With KMT2A/MLL1r, NPM1 Mutations), DLBCL, MM, and CLL/SLL.	NCT05153330
Testing the Addition of an Anti-cancer Drug, SNDX-5613, to the Standard Chemotherapy Treatment (Daunorubicin and Cytarabine) for Newly Diagnosed Patients with Acute Myeloid Leukemia That Has Changes in NPM1 or MLL/KMT2A Gene.	NCT05886049
A Study of BN104 in the Treatment of Acute Leukemia.	NCT06052813
Taken and reported from: https://clinicaltrials.gov/ Accessed: 20 December 2024	
